# An Aldol Reaction-Based Iridium(III) Chemosensor for the Visualization of Proline in Living Cells

**DOI:** 10.1038/srep36509

**Published:** 2016-11-04

**Authors:** Jin-Biao Liu, Li-Juan Liu, Zhen-Zhen Dong, Guan-Jun Yang, Chung-Hang Leung, Dik-Lung Ma

**Affiliations:** 1 Department of Chemistry, Jiangxi University of Science and Technology, Ganzhou, China; 2 Department of Chemistry, Hong Kong Baptist University, Kowloon Tong, Hong Kong, China; 3 State Key Laboratory of Quality Research in Chinese Medicine, Institute of Chinese Medical Sciences, University of Macau, Macao, China

## Abstract

A long-lived aldol reaction-based iridium(III) chemosensor [Ir(ppy)_2_(5-CHOphen)]PF_6_ (**1**, where ppy = 2-phenylpyridine and 5-CHOphen = 1,10-phenanthroline-5-carbaldehyde) for proline detection has been synthesized. The iridium(III) complex **1**, incorporating an aldehyde group in N^N donor ligand, can take part in aldol reaction with acetone mediated by proline. The transformation of the sp^2^-hybridized carbonyl group into a sp^3^-hybridized alcohol group influences the metal-to-ligand charge-transfer (MLCT) state of the iridium(III) complex, resulting in a change in luminescence in response to proline. The interaction of the iridium(III) complex **1** with proline was investigated by ^1^H NMR, HRMS and emission titration experiments. Upon the addition of proline to a solution of iridium(III) complex **1**, a maximum 8-fold luminescence enhancement was observed. The luminescence signal of iridium(III) complex **1** could be recognized in strongly fluorescent media using time-resolved emission spectroscopy (TRES). The detection of proline in living cells was also demonstrated.

Amino acids are core building blocks of living systems, and the detection of amino acids has attracted great interest in fields including chemistry, biochemistry and medicine^1^,^2^,^3^,^4^,^5^,^6^,^7^,^8^. Proline (Pro) is frequently found in β-turn structures of folded protein chains^9^. An excess level of Pro in blood is termed hyperprolinemia (HP-II), which can lead to seizures or intellectual disability^10^,^11^,^12^,^13^. Therefore, the development of highly sensitive and selective Pro detection methods is of great significance.

However, studies regarding luminescent probes for Pro have remain scarce^14^,^15^, which could be due to the weak nucleophilicity and coordination of secondary amino group of Pro compared to primary amino group of other amino acids. Pro has been well documented to catalyze aldol reaction^16^,^17^,^18^, which proceeds *via* an enamine intermediate^19^. Pioneering work by Tanaka, Barbas and co-workers has shown that fluorogenic aldehydes can be used for monitoring aldol reactions *via* fluorescence spectroscopy^20^,^21^, while Kim’s group has developed a coumarin-based aldehyde as an aldol reactant for the selective detection of Pro^15^.

Compared to organic probes^22^,^23^, employing phosphorescent transition metal complexes as chemosensors has several advantages, such as high quantum yields, significant Stokes shifts and long lifetimes which allow them to be potentially used in autofluorescent biological matrices^24^,^25^,^26^,^27^,^28^,^29^,^30^,^31^,^32^,^33^,^34^,^35^,^36^. However, to our knowledge, no previous studies have exploited the detection of Pro in living cells base on the long-lifetime luminescence property of iridium(III) complexes^37^,^38^. Herein, we employed an iridium(III) complex **1**, incorporating an aldehyde group in phenanthroline N^N ligand and two phenylpyridine C^N ligands, as a Pro chemosensor. We anticipated that the metal-to-ligand charge-transfer (MLCT) state of the iridium(III) complex would be influenced by the transformation of sp^2^-hybridized carbonyl group into sp^3^-hybridized alcohol group by Pro-mediated aldol reaction, thereby allowing the complex to work as a luminescent chemosensor for Pro detection (Fig. 1).

## Results

### Photophysical properties of 1

Complex **1** could be conveniently prepared from organometallated dimer [Ir(ppy)_2_Cl]_2_ and 1,10-phenanthroline-5-carbaldehyde (phenald) (Scheme S1, ESI). With the complex **1** in hand, we next investigated the photophysical properties of complex **1**. Complex **1** displays a 3.75 μs lifetime (Table S1, ESI), which is on the same order as phosphorescent iridium(III) complexes, while organic chemosensors generally exhibit lifetimes in nanosecond range. The long-lived luminescence property of transition metal complexes enables them to be detected in highly autofluorescent samples using time-resolved luminescence spectroscopy (TRES), whereby offers them a definite advantage as chemosensors. Moreover, **1** exhibits a maximum emission wavelength at 580 nm upon excitation at 350 nm, with a Stokes shift of approximately 230 nm (Figure S1, ESI), which is much higher than those generally exhibit by organic probes.

### Signal response of 1 to Pro

**Figure 2:**
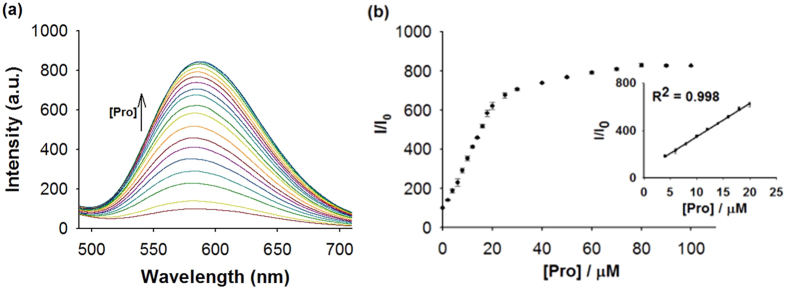
Luminescence emission response of **1** towards Pro. (**a**) Luminescence spectra of **1** (10 μM) with increasing concentration of Pro (0‒10 molar equivalents) in DMSO/acetone (4:1, *v*/*v*). (**b**) Relationship between luminescence intensity and Pro concentration.

**Figure 3:**
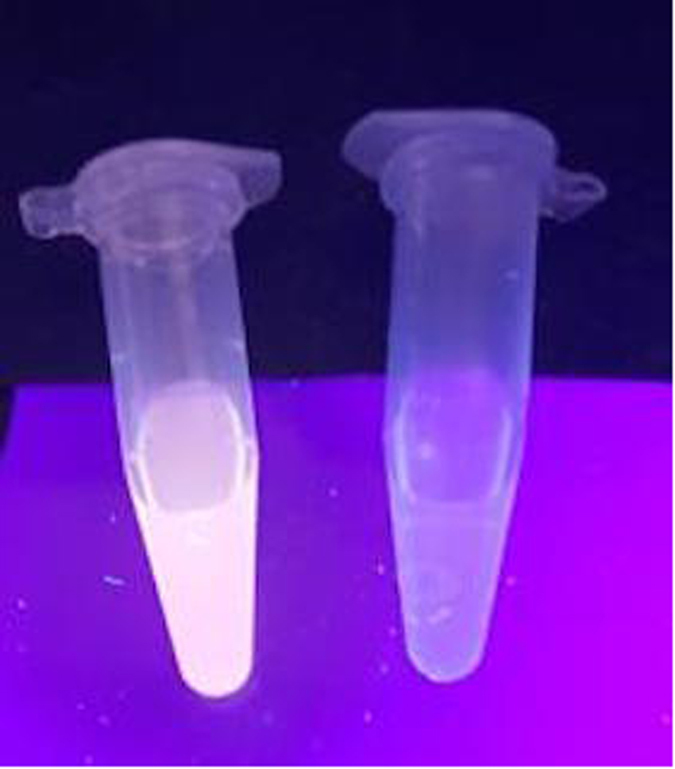
Photographs of **1** (10 μM) with (Left) and without (Right) Pro (100 μM) under UV illumination.

First, we investigated the emission response of **1** towards Pro. In the absence of Pro, the luminescence intensity of **1** was weak in a 4:1 mixture of DMSO and acetone. However, upon addition of Pro, a significant luminescence enhancement of **1** was recorded. Time-course experiments revealed that the luminescence of **1** reached steady-state within 40 min upon addition of Pro (80 μM) at 25 °C (Figure S3, ESI). We next examined the luminescence response of **1** in systems containing different volume ratios of DMSO and acetone. The results indicated that our probe performs best in DMSO/acetone (4:1, v/v), with lower luminescence enhancements being observed when the percentage of acetone solution increases (Figure S4, ESI). In emission titration experiments, the luminescence of **1** (10 μM) enhanced with increasing concentration of Pro and was saturated at ten molar equivalents of Pro, with about an 8-fold enhancement (Fig. 2a). Linear relationship (R^2^ = 0.998) was established with a linear range of 0.4 to 2 molar equivalents of Pro (Fig. 2b), while the limit of detection at a signal-to-noise ratio of 3 was calculated to be 0.75 μM, which is sufficient for detecting Pro in blood that typically contains micromolar levels of Pro^39^. Moreover, the presence of Pro could be observed by naked eyes upon UV illumination using complex **1** (Fig. 3).

### Mechanism validation

**Figure 4:**
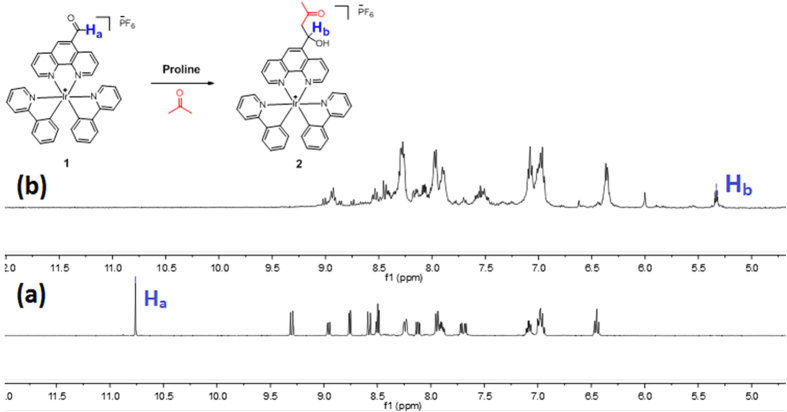
Partial ^1^H NMR spectra of **1** (20 mM) upon the addition of DL-proline (1 molar equivalent) in DMSO-*d*_*6*_/Acetone-*d*_*6*_ (4:1, *v*/*v*). (**a**) **1**. (**b**) **1** + Pro after 4 h.

To verify the mechanism of the assay, the chemical transformation of **1** into aldol product was monitored by ^1^H NMR spectroscopy (Fig. 4). The aldehyde (H_a_ at 10.76 ppm) proton signal of **1** was eliminated upon the addition of DL-Pro to **1** in DMSO-*d*_6_/acetone-*d*_6_ (4:1, *v*/*v*); while a new triplet peak (H_b_ at 5.33 ppm) appeared together with upfield-shifted aromatic protons. These changes are consistent with the formation of the putative aldol product (**2**). Moreover, high-resolution mass spectrometry (HRMS) analysis of the product mixture (with non-deuterated acetone) indicated the formation of product **2** at *m/z* = 767.2455, while the expected signal for complex **1** at *m/z* = 709.2742 was diminished (Figure S5, ESI).

### Selectivity of complex 1 for Pro

**Figure 5:**
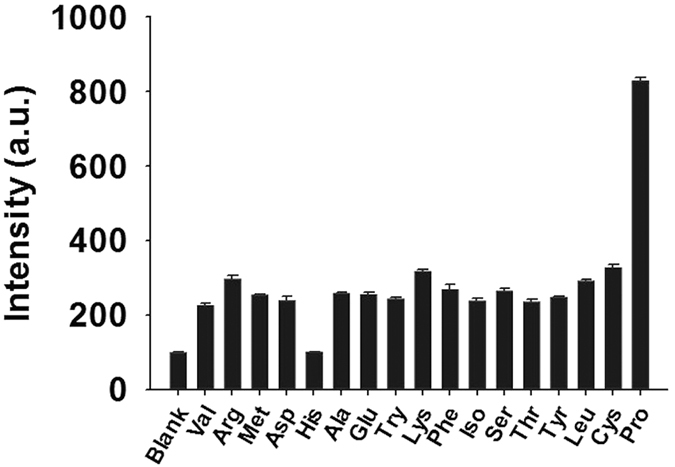
Luminescence response of **1** (10 μM) with 20 molar equivalents of Pro or other amino acids in DMSO/Acetone (4:1, *v*/*v*).

As selectivity is a significant parameter for probe, we evaluated the selectivity of **1** by introducing 20 molar equivalents of Pro or other common amino acids into a solution of **1** (10 μM) (Fig. 5). To our observation, the luminescence response of **1** towards Pro was significantly stronger than that of other amino acids. In order to demonstrate the selectivity of the chemosensor, the tolerance ratio (amount of the interferent that provides 5% of the signal of the analyte) was determined to be 1.03% for lysine, which is the interferent with the strongest luminescence response among other amino acids. This is presumably due to the weaker ability of the primary amines to catalyze aldol reaction versus the secondary amine group of Pro. We also performed a comparative experiment to investigate the response of **1** towards the enantiomers of Pro, and a comparable result was obtained for L, D-Pro and DL-Pro (Figure S6, ESI).

### Time-resolved emission spectra (TRES) of 1

**Figure 6:**
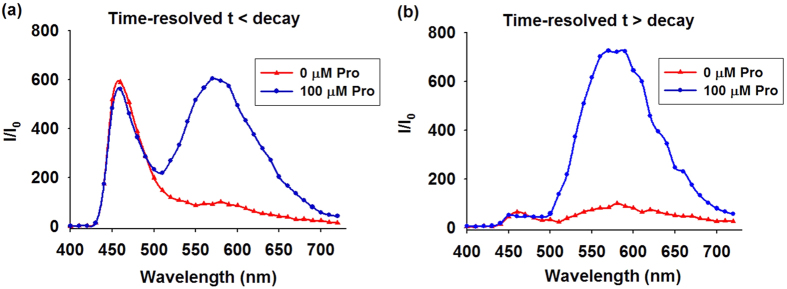
TRES of **1** in the presence of Cm1. (**a**) t < decay. (**b**) t > decay.

To demonstrate that the capability of **1** could be identified in highly fluorescent samples based on its long-lifetime luminescence property via TRES, coumarin 1 (Cm1) was introduced into the system to simulate the autofluorescence environment of biological samples. Cm1 displayed a strong peak at 460 nm region with a tail extended to 600 nm. Consequently, the peak of **1** was significantly perturbed by the emission of Cm1, which would result in an inaccurate determination of Pro concentration (Fig. 6a). To our observation, a distinct peak of **1** was distinguished via TRES when the delay time was defined as post-completion time of Cm1 fluorescence decay. In TRES measurement, no emission peak corresponded to Cm1 was observed, and the spectrum only exhibited the emission peak of **1** (Fig. 6b). These results indicate that the long lifetime luminescence of **1** could potentially be visualized in a strongly autofluorescent biological sample using TRES.

### Application of Pro detection assay in living cells

Considering that living cells are in an aqueous environment, we have also investigated the luminescence response to DMSO-acetone (4:1, v/v) mixed with various percentages of PBS buffer (0–50%). The assay exhibited a gradual reduction in luminescence intensity with increasing proportion of PBS buffer (Figure S7, ESI). Furthermore, the influence of pH value on the assay in DMSO/acetone (4:1, v/v) with 5% PBS was investigated (Figure S8, ESI). The result shows no significant effect of pH on the detection platform in the pH range of 5‒9. Finally, as the ionic strength in the medium increases, the luminescent intensity decreases only slightly (Figure S9, ESI).

**Figure 7:**
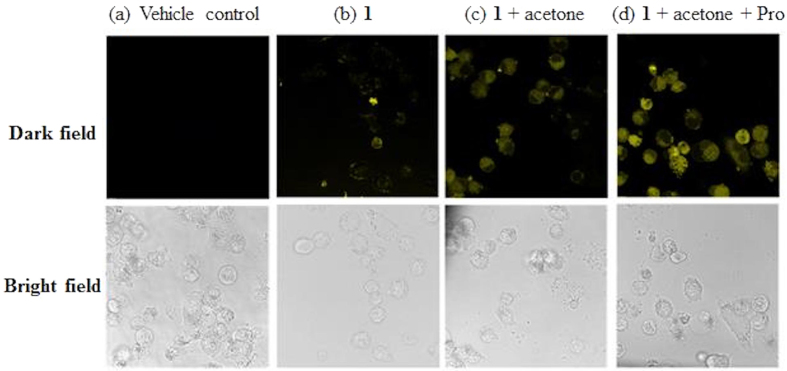
Fluorescence images of A2780 cells. (**a**) Vehicle control in acetone (10 mM). (**b**) Cells were incubated with **1** (10 μM) for 2 h. (**c,d**) Cells were pre-incubated with **1** (10 μM) for 1 h, following by the treatment with (**c**) acetone (10 mM) or (**d**) acetone (10 mM) + Pro (100 μM) for **1** h at 37 °C. The samples were excited at 405 nm with a 63× objective lens.

We next investigated the ability of **1** to visualize Pro in A2780 cells. In the vehicle control experiments, cells were pre-incubated with acetone (10 mM) for 2 h, and no luminescence was observed in the fluorescence images (Fig. 7a). When cells were incubated with only **1** (10 μM) for 2 h, a relatively faint yellow luminescence was observed (Fig. 7b). Once **1** (10 μM) and acetone (10 mM) were present in the culture medium, an obvious yellow luminescence was observed in the cells after 1 h at 37 °C (Fig. 7c). This could possibly be attributed to the catalysis of the aldol reaction by low levels of proline or other secondary amines in the cells. However, after further incubation of cells with Pro (100 μM), the luminescence intensity was significantly increased (Fig. 7d). These results suggest that complex **1** could be taken up into A2780 cells and detect intracellular Pro efficiently.

## Disscusion

In this paper, we have designed and synthesized a successful iridium(III) chemosensor **1** and employed it as a switch-on probe for Pro visualization. **1** contains an aldehyde group in N^N donor ligand, as well as two ppy C^N ligands, which can take part in aldol reaction with acetone mediated by Pro. In the presence of Pro, **1** produced a maximum 8-fold luminescence enhancement at 580 nm. The linear detection range of **1** for Pro was 2‒100 μM and the limit of detection was 0.75 μM. To our knowledge, this is the first iridium(III) switch-on chemosensor for the detection of Pro in living cells. Compared with organic probes, **1** shows a large Stokes shift and a long-lived luminescence allowing it to be distinguished from autofluorescent media via TRES. We anticipate that complex **1** could provide a scaffold for the detection of Pro in biological samples.

## Methods

### Synthesis of 1

A solution of 1,10-phenanthroline-5-carbaldehyde (25.0 mg, 0.12 mmol) and the dichloro-bridged [Ir(ppy)_2_Cl]_2_ (60 mg, 0.056 mmol) in dichloromethane (4 mL) and methanol (4 mL) was stirred at 65 °C overnight^40^,^41^,^42^. After the reaction completed, an excess of solid NH_4_PF_6_ was added and stirred for another 0.5 h at room temperature. The solvent was removed under reduced pressure and the residue was purified by silica gel column chromatography (eluent, methanol/dichloromethane, 1/20, *v*/*v*) to yield **1** as an orange powder.

### Proline detection

1 mM of complex **1** stock solution was prepared in DMSO. The complex was then added into DMSO/acetone (4:1, *v*/*v*) to a final concentration of 10 μM. Different concentrations of Pro were then added to DMSO/acetone containing complex **1** (10 μM) in a cuvette. Luminescence emission spectra were recorded on a PTI QM-4 spectrofluorometer (Photo Technology International, Birmingham, NJ) at 25 °C, with the slits for both excitation and emission set at 2.5 nm (slit width: 1.5 nm; detector voltage: 10 V; delay time: 0.01 ms; gate time: 0.01 ms; excitation: 350 nm; emission: 580 nm; stabilization time: 30 min). UV-Vis absorption spectra were recorded on a Cary UV-300 spectrophotometer (double beam).

### Live cell imaging assay

A2780 cells were seeded at a density of 1 × 106 cells per mL in 35 × 10 mm coverglass-bottom confocal dishes. For vehicle control experiments, acetone (10 mM) solution was pre-incubated with cells for 2 h. For imaging assay, the cells were pre-incubated with **1** (10 μM) for 1 h at 37 °C, followed by washing with PBS buffer three times and further treatment with acetone (10 mM) or with acetone (10 mM) and Pro (100 μM) for 1 h at 37 °C. Fluorescence images of the cells were obtained from Leica TCSSP8 confocal microscope with excitation at 405 mm using a 63× objective lens.

### Photophysical measurement

Emission spectra and lifetime measurements for complexes were performed on a PTI TimeMaster C720 Spectrometer (Nitrogen laser: pulse output 337 nm). Error limits were estimated: λ (±1 nm); τ (±10%); φ (±10%). All solvents used for the lifetime measurements were degassed using three cycles of freeze-vac-thaw.

## Additional Information

**How to cite this article**: Liu, J.-B. *et al*. An Aldol Reaction-Based Iridium(III) Chemosensor for the Visualization of Proline in Living Cells. *Sci. Rep.* **6**, 36509; doi: 10.1038/srep36509 (2016).

**Publisher’s note:** Springer Nature remains neutral with regard to jurisdictional claims in published maps and institutional affiliations.

## Supplementary Material

Supplementary Information

## Figures and Tables

**Figure 1:**
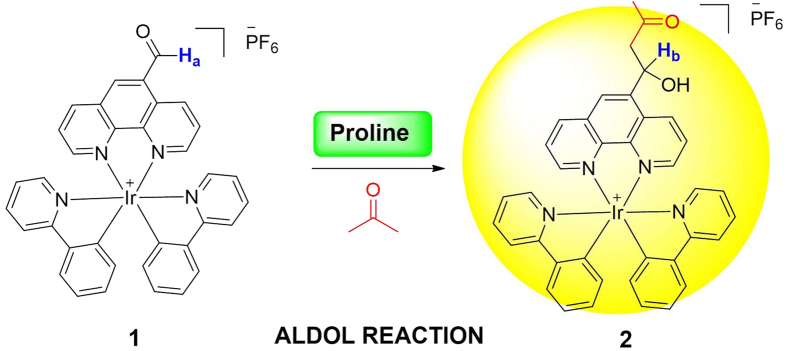
Mechanism of Pro detection by complex **1**.
