# Supplementary material for: An Aldol Reaction-Based Iridium(III) Chemosensor for the Visualization of Proline in Living Cells
Source: Sci Rep. 2016 Nov 4;6:36509. doi: 10.1038/srep36509 (PMC5095674; doi:10.1038/srep36509)
Supplement: Supplementary Information [file srep36509-s1.pdf]

Supporting Information

An Aldol Reaction-Based Iridium(III) Chemosensor for the Visualization of Proline in Living Cells

Jin-Biao Liu[a,b,*], Li-Juan Liu[c], Zhen-Zhen Dong[b], Guan-Jun Yang[c], Chung-Hang Leung[c] and Dik-Lung Ma[b,*]

[a] Department of Chemistry, Jiangxi University of Science and Technology, Ganzhou, China.
[b] Department of Chemistry, Hong Kong Baptist University, Kowloon Tong, Hong Kong, China.
[c] State Key Laboratory of Quality Research in Chinese Medicine, Institute of Chinese Medical Sciences, University of Macau, Macao, China.
* Corresponding author:
Dr. Jin-Biao Liu, E-mail: liujbgood@hotmail.com.
Dr. Dik-Lung Ma, E-mail: edmondma@hkbu.edu.hk, Tel: (+852) 3411-7075, Fax: (+852) 3411-7348.

Contents:

General experimental. 2
Materials. 2
Scheme S1. Synthesis of **1**. ..... 2 & 3
Table S1. Photophysical properties of iridium(III) complex **1**. 4
Figure S1. Excitation and emission spectra of **1** (10 μM) in DMSO/acetone (4:1, *v*/*v*). 4
Figure S2. UV-Vis absorption spectra of **1** (20 μM) in DMSO 4
Figure S3. Time course of luminescence response of **1** (10 μM) in the presence of Pro (80 μM) at 25 °C. 5
Figure S4. Luminescence enhancement of **1** (10 μM) with Pro (80 μM) in various percentage of acetone in DMSO. 5
Figure S5. High-resolution mass spectrum of the reaction product **2**. 5
Figure S6. Luminescence enhancement of system in response to **1** (10 μM) in the absence or presence of L, D, or DL-proline (80 μM). 6
Figure S7. Luminescence enhancement of **1** (10 μM) with Pro (80 μM) in various percentage of PBS buffer in DMSO/acetone (4:1, *v*/*v*). 6
Figure S8. Influence of pH (5 to 9) on the luminescence enhancement of **1** (10 μM) with Pro (80 μM) in the assay of 5% PBS medium. 6
Figure S9. Influence of KCl concentration on the luminescence enhancement of **1** (10 μM) with Pro (80 μM) in the assay of 5% PBS medium. 7

General experimental. Mass spectrometry was performed at the Mass Spectroscopy Unit at the Department of Chemistry, Hong Kong Baptist University, Hong Kong (China). Deuterated solvents for NMR purposes were obtained from Armar and used as received.

^{1}H and ^{13}C NMR were recorded on a Bruker Avance 400 spectrometer operating at 400 MHz (^{1}H) and 100 MHz (^{13}C). ^{1}H and ^{13}C chemical shifts were referenced internally to solvent shift (acetone-d_6: ^{1}H, δ 2.09, ^{13}C δ 205.87, 30.60; DMSO-d_6: ^{1}H δ 2.50, ^{13}C δ 39.5). Chemical shifts (δ) are quoted in ppm, the downfield direction being defined as positive. Uncertainties in chemical shifts are typically ± 0.01 ppm for ^{1}H and ± 0.05 for ^{13}C. Coupling constants are typically ± 0.1 Hz for ^{1}H-^{1}H and ± 0.5 Hz for ^{1}H-^{13}C couplings. The following abbreviations are used for convenience in reporting the multiplicity of NMR resonances: s, singlet; d, doublet; t, triplet; q, quartet; m, multiplet; br, broad.

Materials. Reagents, unless specified, were purchased from Sigma Aldrich (St. Louis, MO) and used as received. Iridium chloride hydrate ($IrCl_3 \cdot xH_2O$) was purchased from Precious Metals Online (Australia).

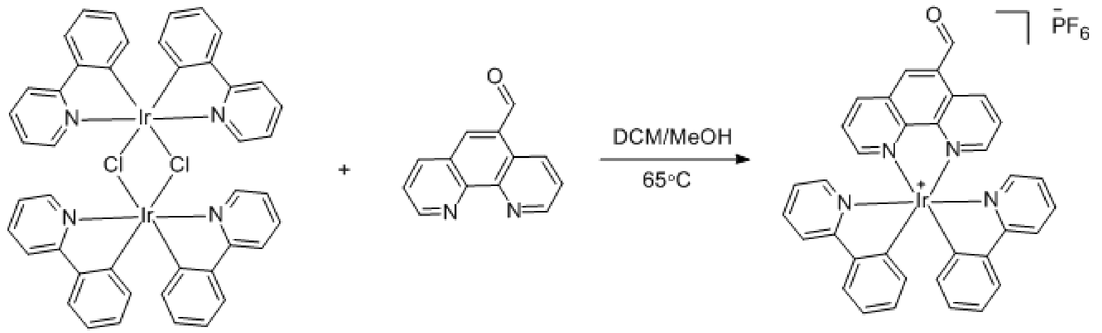

Scheme S1. Synthesis of **1**.

Complex **1**. Yield: 64%. ^{1}H NMR (400 MHz, acetone) δ 10.76 (s, 1H), 9.30 (d, *J* = 9.2 Hz, 1H), 8.95 (dd, *J* = 8.3, 1.3 Hz, 1H), 8.76 (d, *J* = 5.1 Hz, 1H), 8.58 (d, *J* = 9.2 Hz, 1H), 8.50 (dd, *J* = 6.6, 4.5 Hz, 2H), 8.25 – 8.23 (m, 1H), 8.12 (dd, *J* = 8.3, 5.1 Hz, 1H), 7.93 (d, *J* = 1.7 Hz, 2H), 7.92 – 7.87 (m, 2H), 7.70 (dd, *J* = 15.6, 5.5 Hz, 2H), 7.08 (tdd, *J* = 7.7, 3.5, 1.1 Hz, 2H), 7.00 – 6.93 (m, 4H), 6.45 (t, *J* = 7.7 Hz, 2H); ^{13}C NMR (100 MHz, acetone) δ 192.95, 168.65, 168.53, 153.23, 152.72, 150.44, 149.01, 147.60, 145.20, 145.05, 139.64, 139.08, 132.70, 132.40, 131.59, 131.33, 130.92, 129.33, 128.45, 125.88, 125.40, 124.47, 123.69, 120.85. MALDI-TOF-HRMS: Calcd. for $C_{38}H_{32}F_6IrN_4OP$ $[M–PF_6]^+$: 709.1592, found: 709.1576.

^{1}H NMR, ^{13}C NMR and mass spectrum of **1**.

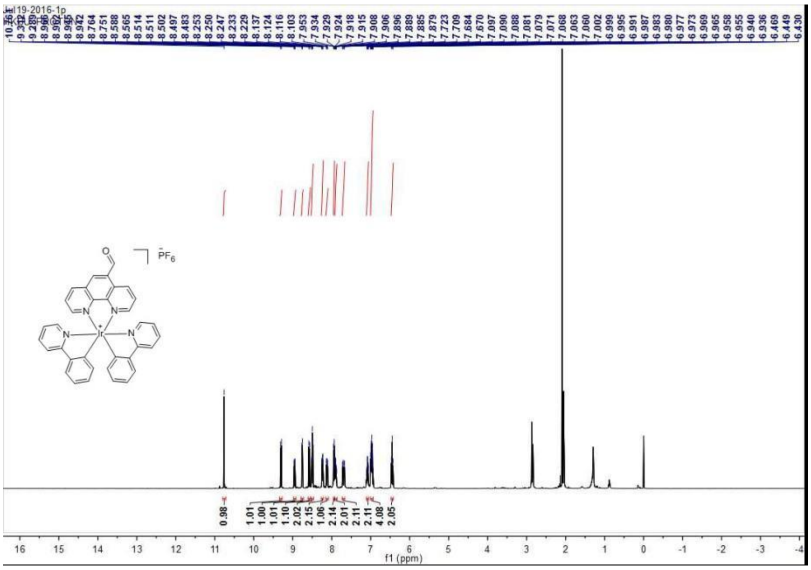

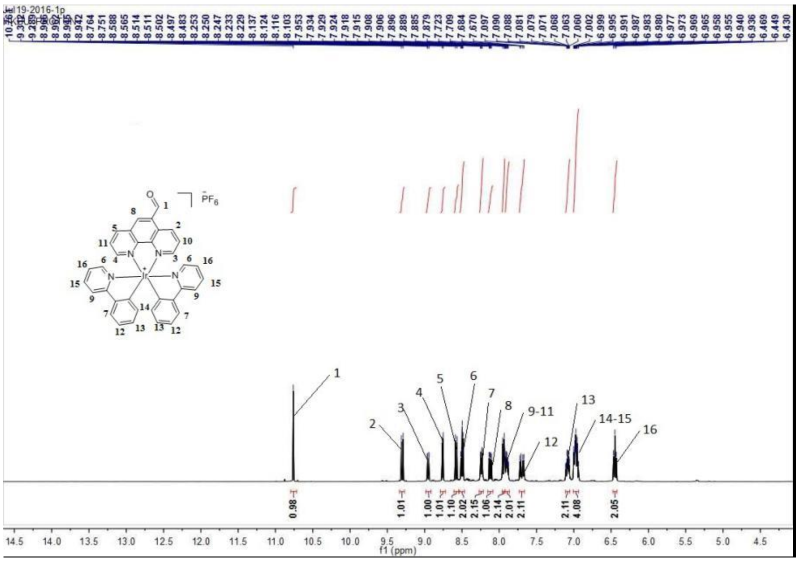

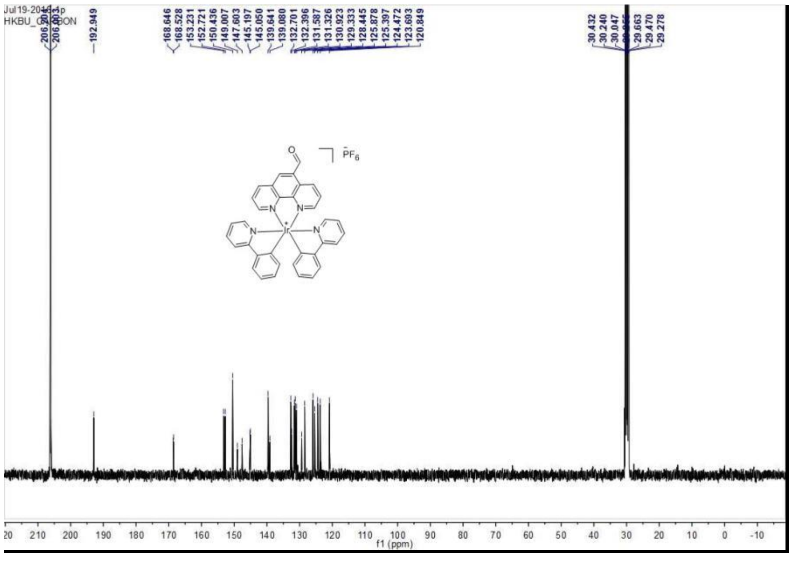

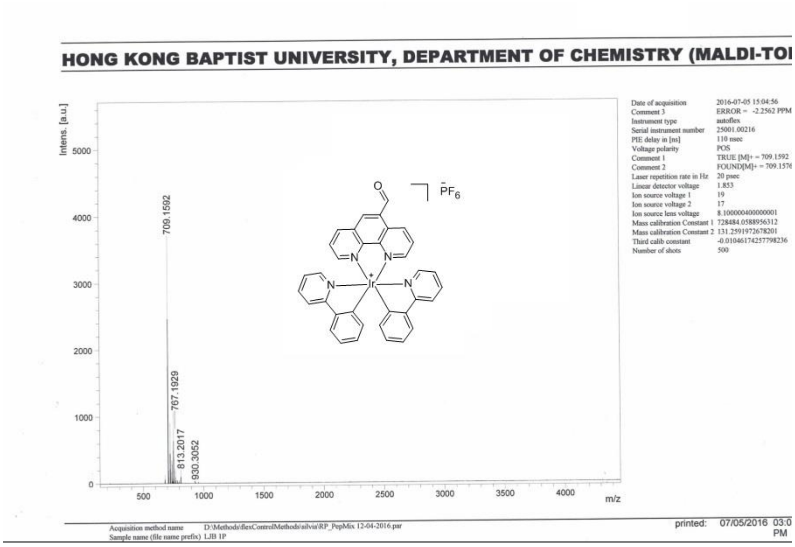

Table S1. Photophysical properties of iridium(III) complex **1**.

Quantum yield	λ_{em}/ nm	Lifetime/ μs	UV-Vis absorption λ_{abs}/ nm (ε/ dm^3 mol^{-1} cm^{-1})
0.718	580	3.750	235 (3.6×10^4), 370 (1.5×10^3)

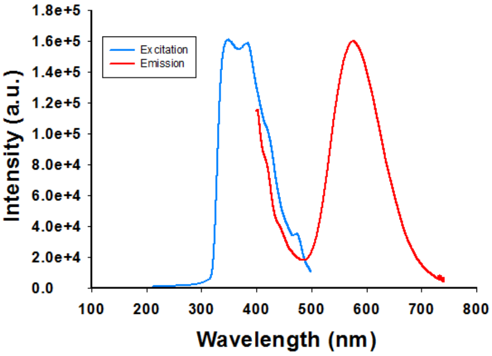

Figure S1. Excitation and emission spectra of **1** (10 μM) in DMSO/acetone (4:1, *v*/*v*).

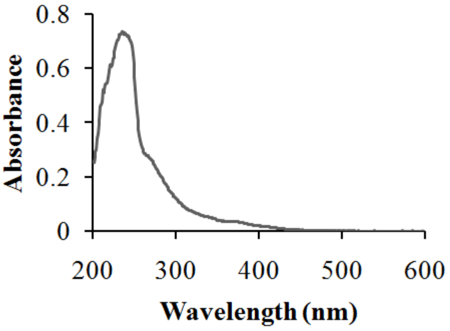

Figure S2. UV-Vis absorption spectra of **1** (20 μM) in DMSO.

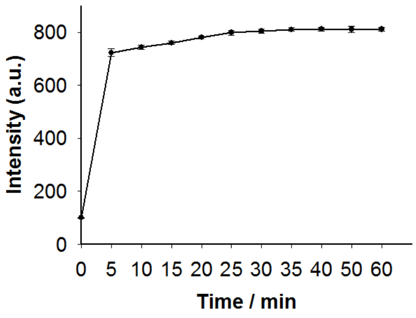

Figure S3. Time course of luminescence response of **1** (10 μM) in the presence of Pro (80 μM) at 25 °C.

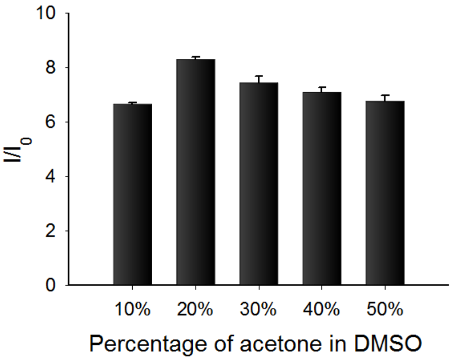

Figure S4. Luminescence enhancement of **1** (10 μM) with Pro (80 μM) in various percentage of acetone in DMSO.

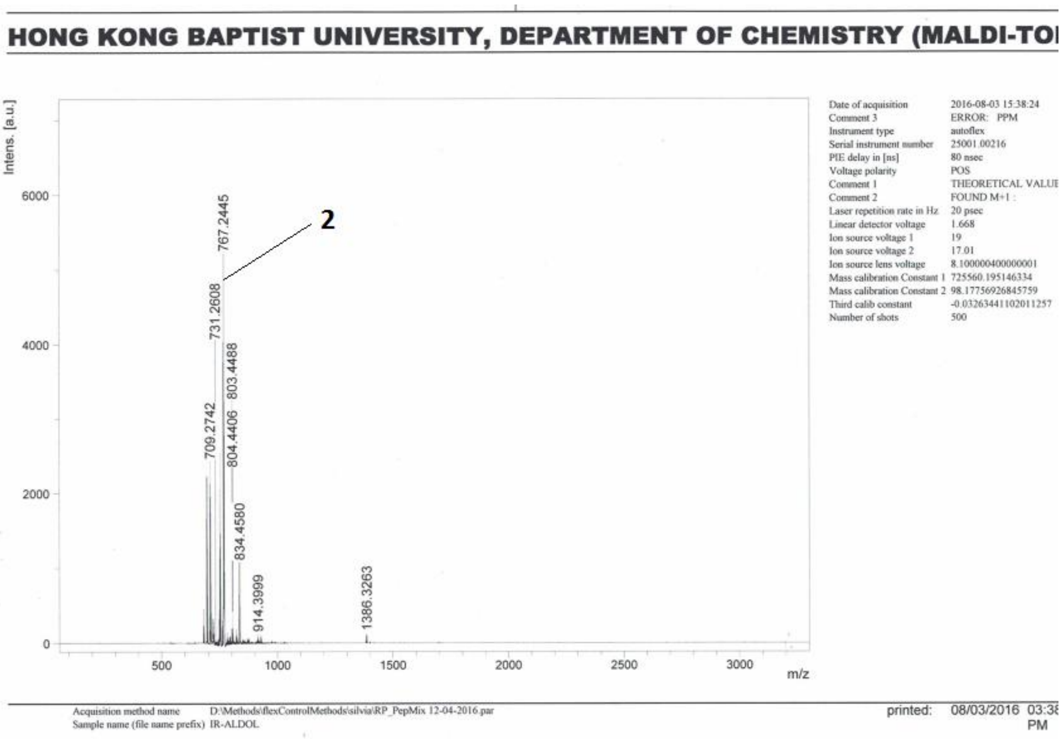

Figure S5. High-resolution mass spectrum of the reaction product **2**.

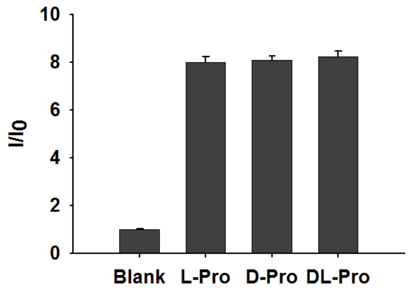

Figure S6. Luminescence enhancement of system in response to **1** (10 μM) in the absence or presence of L, D, or DL-proline (80 μM).

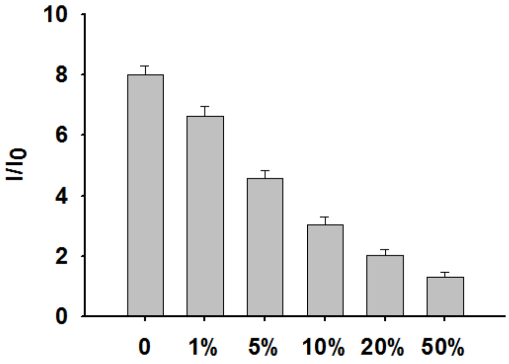

Figure S7. Luminescence enhancement of **1** (10 μM) with Pro (80 μM) in various percentage of PBS buffer in DMSO/acetone (4:1, *v*/*v*).

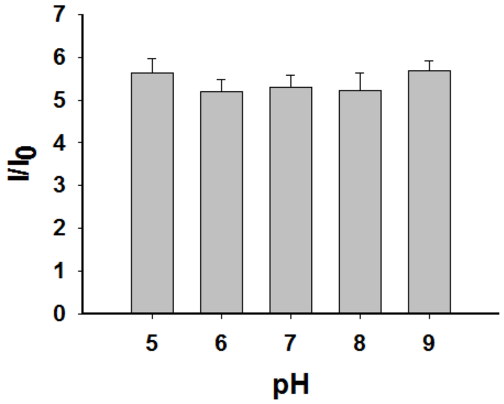

Figure S8. Influence of pH (5 to 9) on the luminescence enhancement of **1** (10 μM) with Pro (80 μM) in DMSO/acetone (4:1, *v*/*v*) with 5% PBS medium.

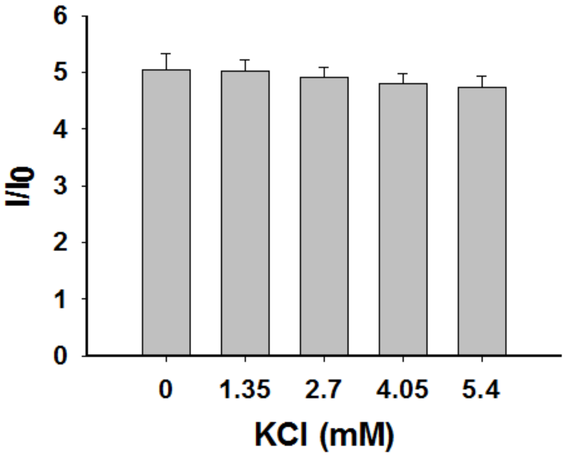

Figure S9. Influence of KCl concentration on the luminescence enhancement of **1** (10 μM) with Pro (80 μM) in DMSO/acetone (4:1, *v*/*v*) with 5% PBS medium.
